# Novel 1,3,4-oxadiazole-nicotinamide hybrids as non-classical AHL mimics quorum sensing inhibitors of *Pseudomonas aeruginosa*: design, synthesis and biological evaluation

**DOI:** 10.1186/s13065-025-01560-9

**Published:** 2025-07-11

**Authors:** Mohamed M. S. Hamoud, Amany M. Ghanim, Abdalla E. A. Hassan, Hanan A. Abdel-Fattah, Hisham A. Abbas, Noura M. Seleem, Hend Kothayer, Nermine A. Osman

**Affiliations:** 1 Department of Pharmaceutical Organic Chemistry, Faculty of Pharmacy, Zagazig University, Zagazig, 44519 Egypt; 2 Department of Chemistry, Faculty of Science, Zagazig University, Zagazig, 44519 Egypt; 3 Department of Microbiology and Immunology, Faculty of Pharmacy, Zagazig University, Zagazig, 44519 Egypt; 4 Department of Medicinal Chemistry, Faculty of Pharmacy, Zagazig University, Zagazig, 44519 Egypt

**Keywords:** Quorum sensing, *Pseudomonas aeruginosa*, Anti-virulence, Nicotinamide, Oxadiazole, Modelling

## Abstract

*Pseudomonas aeruginosa* employs *N*-acylated L-homoserine lactones (AHLs) as autoinducers (AIs) for quorum sensing, which in the end is responsible for biofilm and virulence factor production along with the development of antibiotic resistance in bacteria. Here, we designed and synthesized a library of *S*-alkyl-1,3,4-oxadiazoles bearing nicotinamide moiety targeting AHLs receptors (LasR and RhlR) and evaluated their efficacy as new inhibitors of quorum sensing. The minimum inhibitory concentrations of the synthesized compounds against *P. aeruginosa* PAO1 were determined, and they ranged from 2.5 to 5 mg/ml. All the compounds were subjected to further investigation against protease and pyocyanin. The oxadiazole derivative **3a** was the most potent compound that reduced both protease activity and pyocyanin production by 38.46% and 70.27% respectively. Moreover, compound **3a** showed a significant reduction of the biofilm formation by 81.72%. A docking study was performed to explore the potential binding interactions with quorum-sensing receptors (LasR and RhlR), which are responsible for the expression of virulence genes. Molecular docking results indicated that compound **3a** showed a comparable binding affinity to the co-crystalized ligands of two *P. aeruginosa* QS targets (lasR and RhlR) with higher affinity towards LasR than RhIR active sites. Our findings highlight the promising potential of 1,3,4-oxadiazole-nicotinamide hybrid **3a** as an anti-virulence agent to combat *P. aeruginosa*.

**Graphical Abstract:**

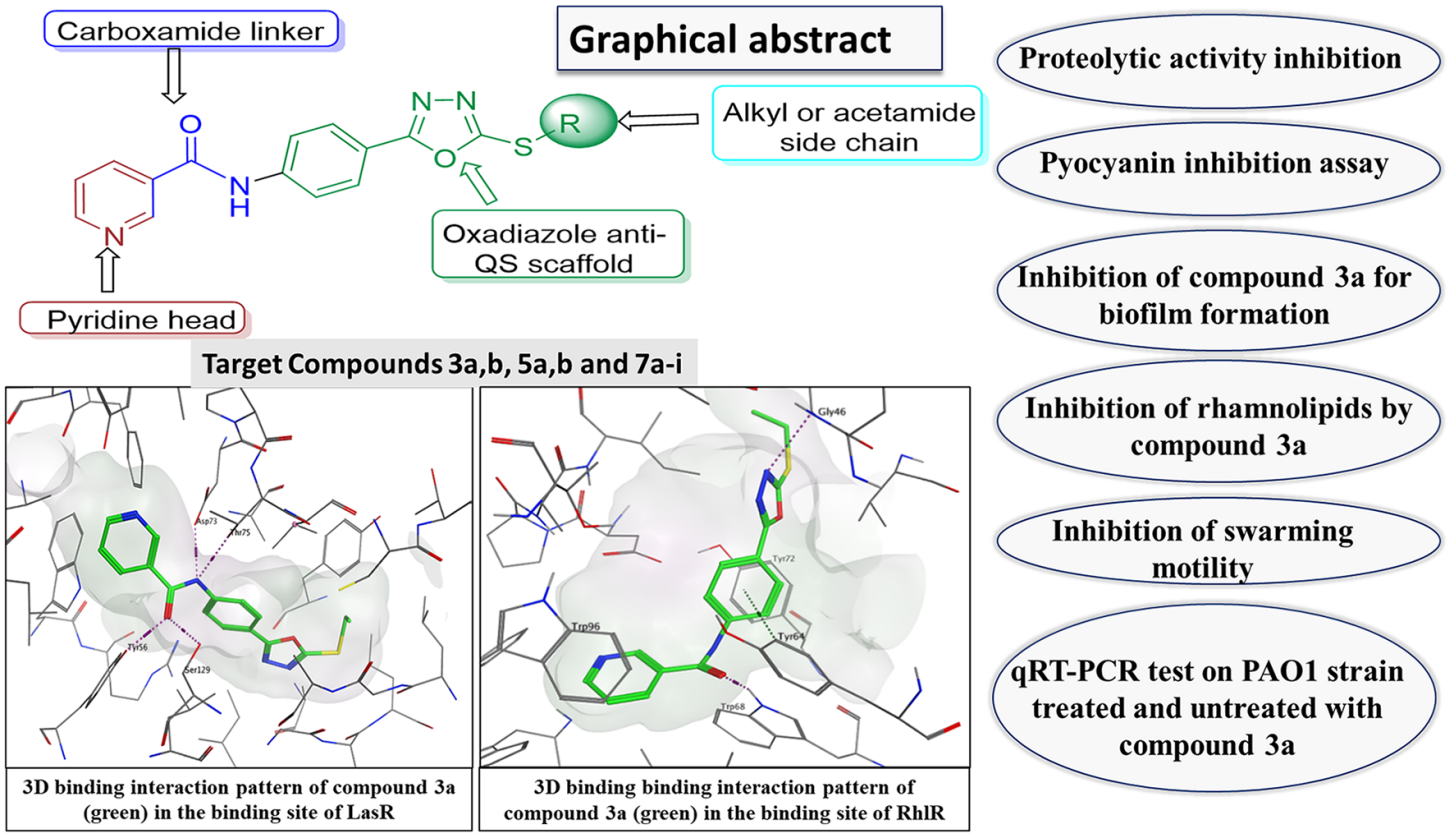

## Introduction

*Pseudomonas aeruginosa* is a frequent etiological agent of healthcare associated infections that commonly affects patients with weak immunity [1]. *P. aeruginosa* can result in infections of the urinary tract and the respiratory tract. Also, it can contaminate skin with wounds or burns [2]. To treat *P. aeruginosa* infections, there is a great challenge for public health workers represented by its high resistance to antimicrobial agents. To combat antimicrobial resistance, attacking bacterial virulence regulated by quorum sensing system (QS) machinery [3, 4]. QS is a communication machinery for bacterial cells. The communicative machinery is cell number dependent. The sensing of bacterial numbers is based upon the release of autoinducers or signaling molecules termed N-acylated homoserine lactones (AHLs). When the quorum of autoinducers is achieved, the autoinducers bind to their receptors leading to altered virulence genes expression [5]. QS systems include LasI-LasR, RhlI-RhlR, and PQS-MvfR. Concerning LasI-LasR system, *N*-(3-oxododecanoyl)-L-homoserine binds to LasR receptor. On the other hand, in RhlI-RhlR system, *N*-butyryl-L-homoserine lactone binds with RhlR receptor, while in PQS-MvfR system, 2-heptyl-3-hydroxy-4(1*H*) quinolone (PQS) is the autoinducer [6–8]. Targeting QS is advantageous in terms of the low likelihood of the emergence of resistance due to the absence of killing bacterial cells and keeping beneficial gut flora [9–11]. Therefore, one of the most important challenges is to synthesize and develop new potent and broad-spectrum antimicrobial agents that might disrupt microbial resistance.

**Fig. 1:**
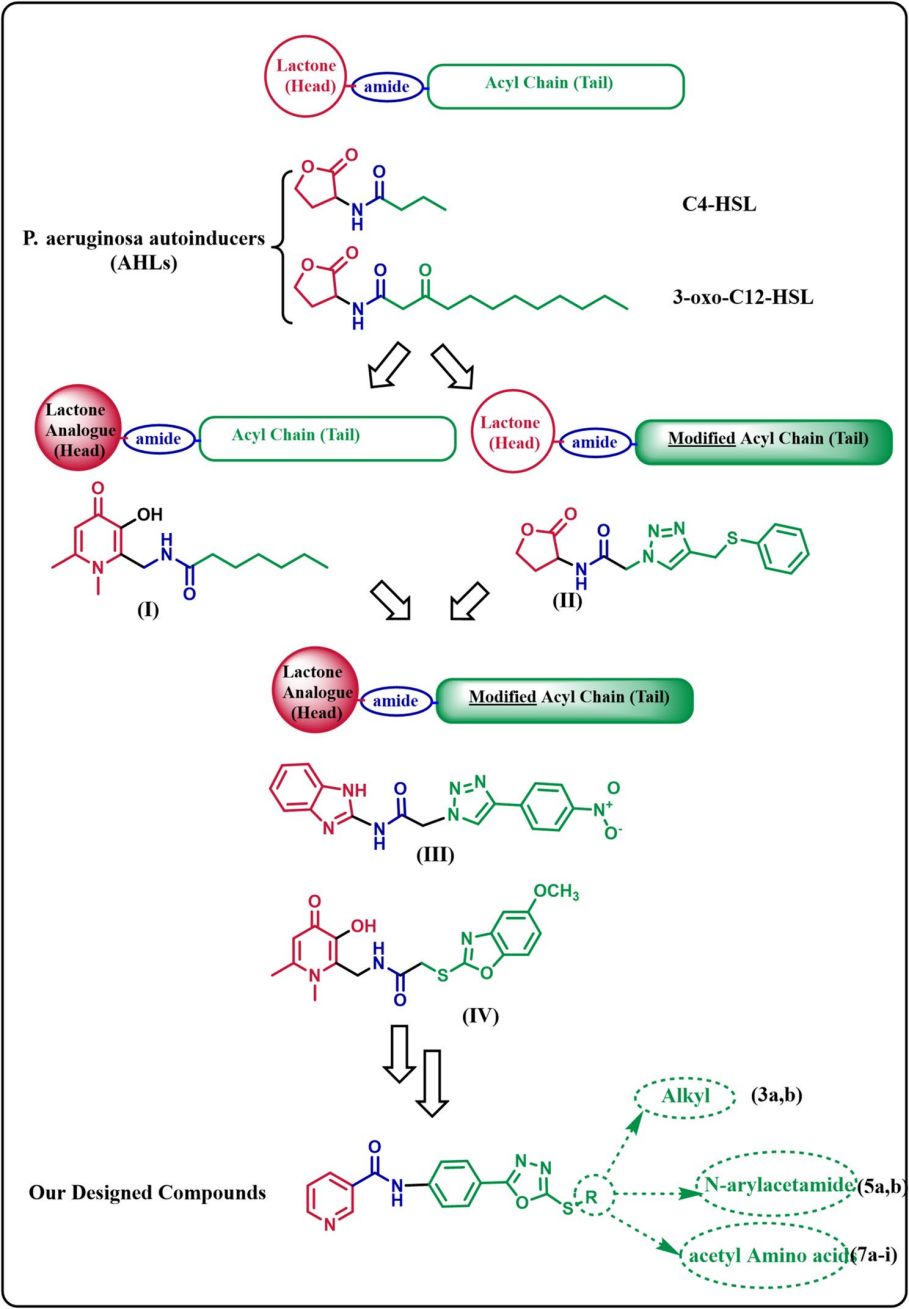
*P. aeruginosa* autoinducers (AHLs) and their structural features, some reported AHL analogues (I-IV), and rationale for the design of our compounds **3a**,** b**,** 5a**,** b** and **7a-i**

Several quorum-sensing inhibitors (QSIs) were designed as AHL analogues to compete with the auto-inducers and subsequently block the *P. aeruginosa* QS signals [12–14]. AHL scaffold is composed of three features: the head (the homoserine lactone), the tail (a linear *N*- acyl chain) and the amide bridge between both segments [14, 15], (Fig. 1). AHL structural modifications were carried out on either the head, the tail or both [12–14],. As the lactone head is subjected to activity loss either *via* alkaline ring opening or enzymatic degradation by other bacteria or hosts lactonases [16, 17],, it was successfully replaced by other stable rings as cyclopentanone, quinoline and tetrahydrofuran [12–14],. Recent literature has reported a QS inhibitor with 4-oxo-1,4-dihydropyridine head **(I)** that exhibited 68.67% *P. aeruginosa* biofilm inhibitory activity at 20 µM [18] (Fig. 1).

On the other side, many reported AHL analogues keep the lactone head group with a modified acyl chain where the tail may incorporate one or two phenyl rings, O, S and/or heterocyclic ring [12–14],. As the LasR AHL-binding pocket is highly flexible and can accommodate variable ligands, sometimes the tail has been drastically modified [15], exemplified by the sulfur-modified triazole-containing AHLs **(II)** [19] (Fig. 1). Modification of both the head and the tail was also successful in developing bioactive QS inhibitors [12–14]. For example, the 1,2,3-triazole-based 2-aminobenzimidazole **(III)** exhibited QS inhibitory activity against *P. aeruginosa* at low concentrations with 63.67% inhibition at 62.5 mM [20]. Most recently, 3-hydroxypyridin-4(1*H*)-ones based hybrids **(IV)** showed *P. aeruginosa* biofilm inhibitory activity with IC_50_: 10.59 ± 1.17 µM [21].

**Fig. 2:**
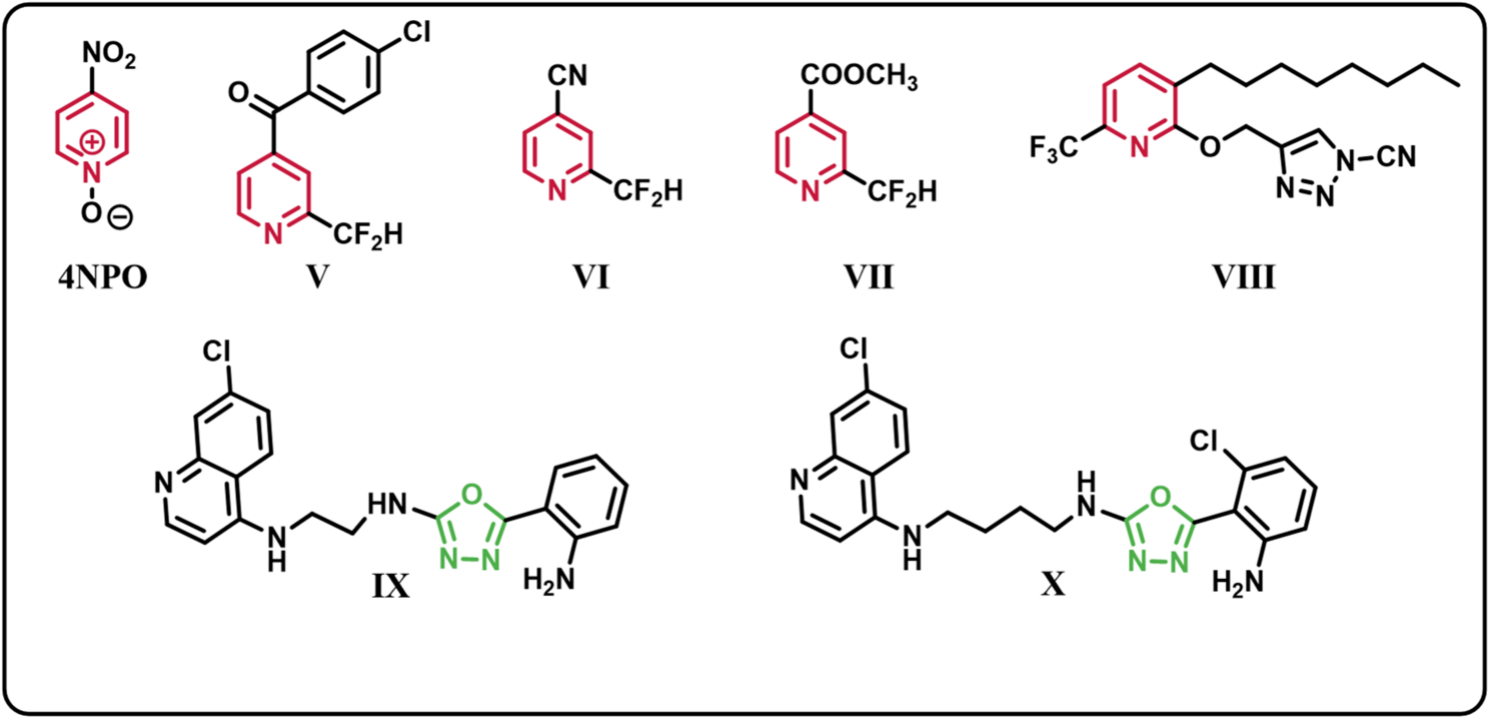
Some reported pyridine containing QS inhibitors **(4NPO** and **V-VIII)**, and some anti-QS 1,3,4-oxadiazoles **(IX** and **X)**

In our design, we adopted the “head and tail” modification strategy, where we chose the pyridine ring as the head because it has the ability to keep hydrogen-bonding interactions similar to the lactone ring and being presented in many reported QS inhibitors **(4NPO** and **V-VIII)** [22, 23] (Fig. 2). In the tail, we incorporated oxadiazole as a promising anti-QS scaffold that exhibited significant anti-virulence activity against *P. aeruginosa* as in compounds **IX** and **X** [24] (Fig. 2). Besides oxadiazole, S and phenyl moieties were also included in the tail segment to increase interactions with the receptor and potentially discover additional opportunities to improve binding (Fig. 1). To expand SAR study, we made an extension with either alkyl, *N*-arylacetamides or acetyl amino acids to study the effect of these variations on the activity. The rationale of this study is the use of structural analogues of the natural autoinducers acylhomoserine lactones as competitive inhibitors of binding of the autoinducers to their cognate receptors. Furthermore, a previous study showed the ability of 1,3,4 oxadiazole hybrids to inhibit QS in *P. aeruginosa* PAO1. Moreover, nicotinamide could inhibit biofilm formation and virulence of *Streptococcus mutans* [25].

The reported significant QS inhibitory activity of the 1,3,4 oxadiazole and nicotinamide [22–24] inspired us to combine both pharmacologically active moieties and design novel 1,3,4-oxadiazole-nicotinamide hybrids mimicking the natural autoinducers *N*-acylated homoserine lactones, where the nicotinamide was used as the head while 1,3,4-oxadiazole was incorporated in the tail for the purpose of achieving synergistic QS inhibitory activity. Moreover, we incorporated an extension of S and phenyl with either alkyl, *N*-arylacetamides or acetyl amino acids. These structural modifications could increase QS inhibition due to spatial changes and could help to explore further possible target interactions. Hoping to develop more potent QS inhibitors and subsequently, enhance their efficacy against *P. aeruginosa* since *P. aeruginosa* mutants without a functional QS system are unable to develop into mature biofilms and are non-virulent [14].

In this study, the generated compounds were first pre-screened for the inhibitory activity against protease and pyocyanin production in *P. aeruginosa* (PAO1) standard strain using sub-inhibitory concentrations. Then the most active compound **3a** was further investigated for the anti-virulence/ anti-QS activities and in-silico analysis to assess its binding affinity to QS receptors in *P. aeruginosa*.

## Results and discussion

### Chemistry

The formation of the target *S*-alkylated oxadiazole derivatives **3a**,** b**, **5a**,** b** and **7a-i** was achieved starting from the reported compound *N*-(4-(hydrazinecarbonyl) phenyl) nicotinamide **(1)** [26]. 5-Thioxo-1,3,4-oxadiazole **2** was obtained in a very good yield from the reaction of the carboxylic acid hydrazide **1** with carbon disulphide in ethanol containing potassium hydroxide under reflux [27]. Compound **2** was subjected to *S*-alkylation using different alkylating agents at different reaction conditions to afford the target compounds. For example, diethyl sulphate in aqueous potassium carbonate at room temperature, for compound **3a**. Isopropyl bromide in ethanol containing potassium hydroxide at room temperature for compound **3b**. However, compounds **5a**,** b** and **7a-i** were delivered using the previously synthesized 2-chloro-*N*-phenylacetamide derivatives **4a**,** b** and *N*-(2-chloroacetyl) amino acid derivatives **6a-i** respectively in acetone containing potassium carbonate adopting the reported procedure [27].

**Fig. 3:**
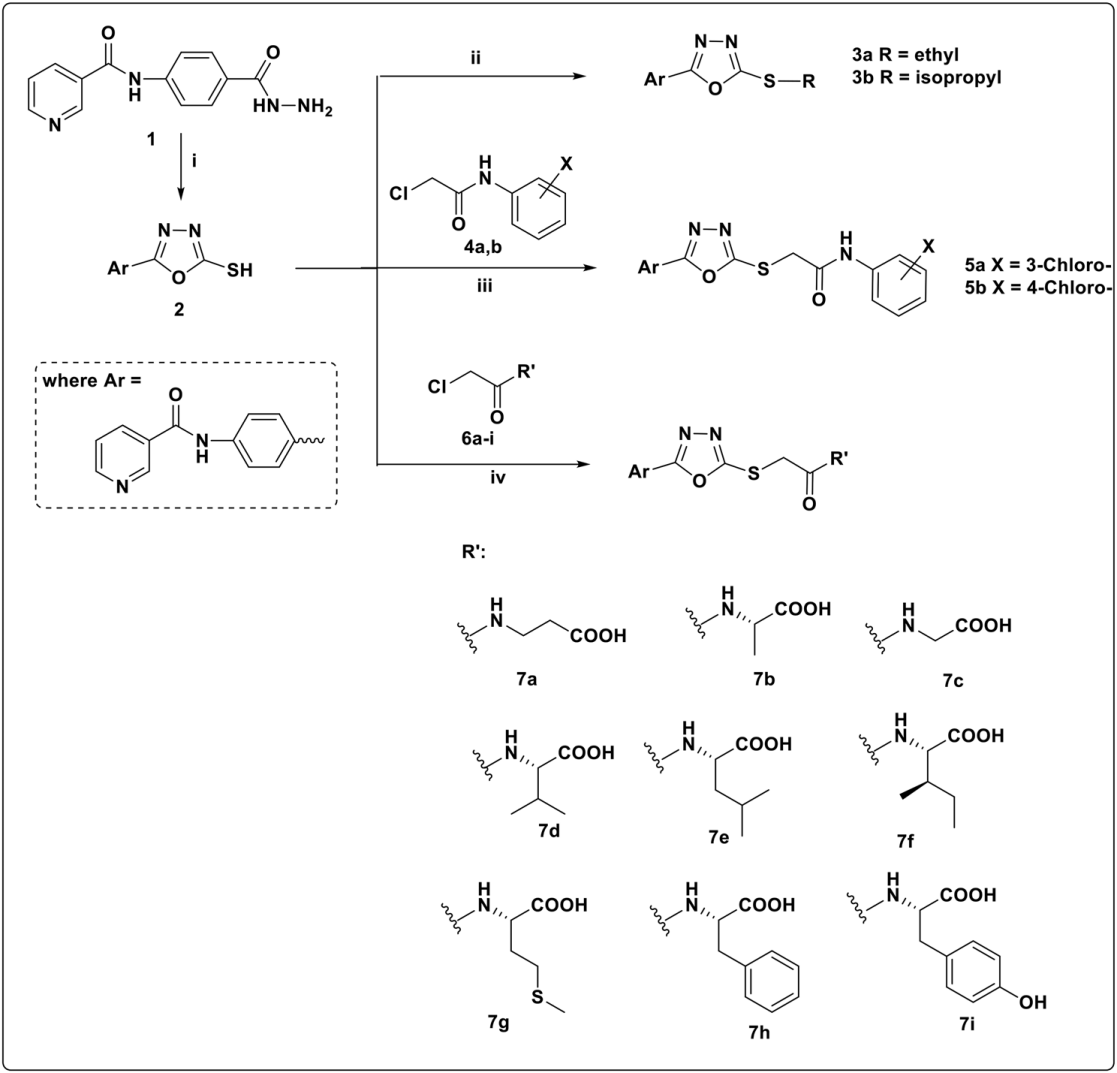
Synthesis of oxadiazole derivatives 3a, b, 5a, b and 7a-i. Reagents and conditions; (i) CS_2_, KOH, EtOH, reflux, 15 h. (ii) (C_2_H_5_)_2_SO_4_, K_2_CO_3_, H_2_O, rt, 1 h. for 3a and (CH_3_)_2_CHBr, EtOH, KOH, rt, 24 h. for 3b. (iii) 2-Chloro-*N*-phenylacetamide derivatives, acetone, K_2_CO_3_, reflux. (iv) *N*-(2-Chloroacetyl) amino acids (6a-i), acetone, K_2_CO_3_, reflux

### Microbiology

#### Minimum inhibitory concentration (MIC) of compounds against *P. aeruginosa* PAO1

*P. aeruginosa* PAO1 growth was inhibited at concentrations (MIC) of 2.5-5 mg/ml. Then, 1/8 MIC of each compound was used to screen for the inhibitory activity against protease and pyocyanin. Herein, the MIC values were much smaller than those of Ghameshlouei et al. [28]., who reported MIC values of ≤ 125 and ≤ 1000 mg/ml for the 1, 3, 4-oxadiazole compounds against different *P. aeruginosa* strains.

#### Proteolytic activity inhibition

**Fig. 4:**
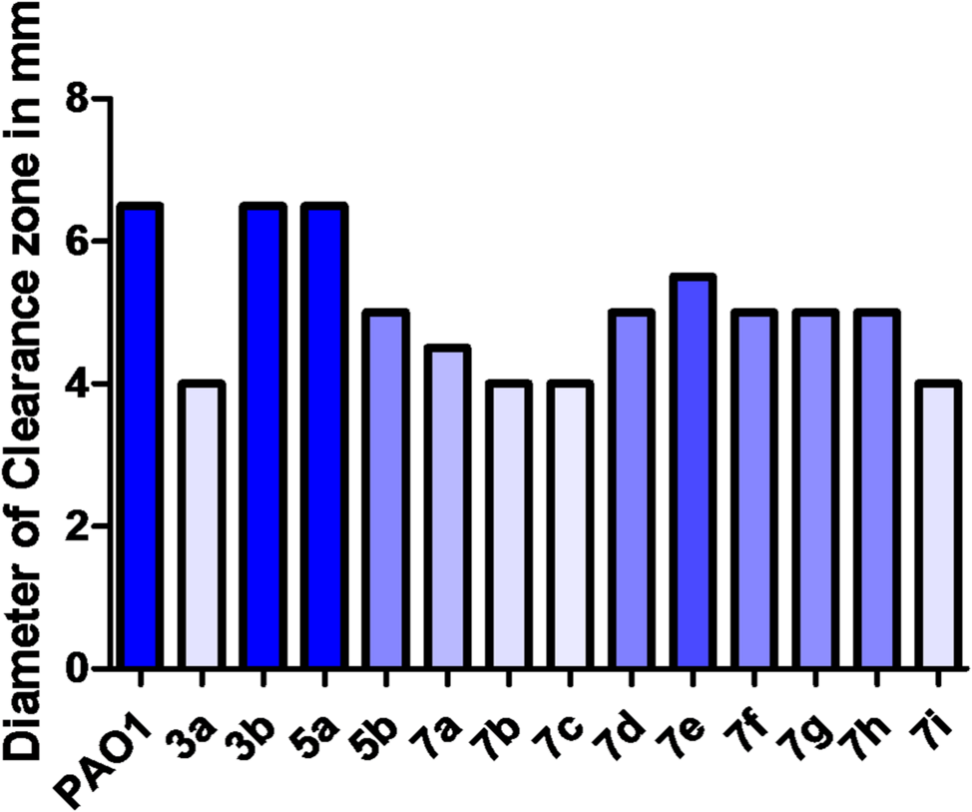
Inhibitory effect of compounds against protease activity in *P. aeruginosa* (Diameter in mm). Compounds **3a**,** 7b**,** 7c** and **7i** showed the highest anti-proteolytic activity

Protease has a well-established role in Pseudomonal infections, as it can destroy immune components protecting mucous membranes and enhance tissue damage [29]. The skim milk agar method was used for protease inhibition screening. The tested compounds showed variable results against protease activity. The *S*-ethyl oxadiazole derivative 3a and the amino acids derivatives 7b, 7c, and 7i reduced activity by 38.46% while the β-alanine derivative 7a decreased the activity by 30.77%. These oxadiazole derivatives were the most active compounds as they showed the narrowest clear zones (Fig. 4).

### Pyocyanin inhibition assay

**Fig. 5:**
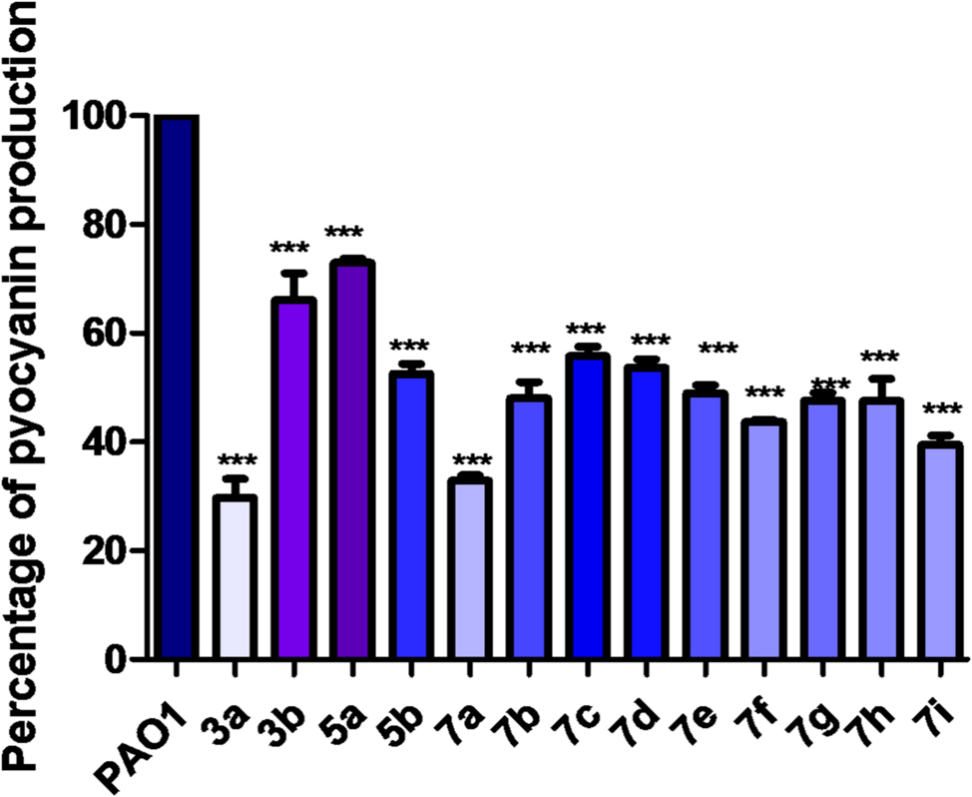
Inhibitory effect of compounds against pyocyanin in *P. aeruginosa*. All compounds significantly reduced pyocyanin production, but compound **3a** was the most effective inhibitor of pyocyanin. (All data are representative of three independent experiments performed in triplicate and expressed as the mean ± SD values in each bar. * *p* < 0.05). One Way ANOVA test followed by Dunnett multiple comparison test was used for statistical analysis

Pyocyanin (PCN) is a characteristic pigment that *P. aeruginosa* produces as a secondary metabolite. Through an eDNA-related mechanism, PCN promoted biofilm formation. Also, it mediates host cell damage by altering their redox balance [30]. In this test, pyocyanin pigment was reduced by all compounds but to different extents. The most active compound among the examined oxadiazole series was the ethyl derivative 3a which decreased pyocyanin production by 70.27%. Despite the mild anti-pyocyanin activity of amino acids’ series 7a-i, the β*-*alanine derivative 7a showed superior efficacy with 67.16% inhibition (Fig. 5). Comparable to our findings, Zender et al.., have reported an oxadiazole-2-amine derivative as an antagonist of PqsR, an Important Regulator of *P. aeruginosa* virulence, and it remarkably reduced the level of pyocyanin in wild-type *P. aeruginosa* [31]. Another study showed that one of the synthesized 2,5-disubstituted 1,3,4-oxadiazoles diminished the production of pyocyanin by more than 70% in comparison to the control cells [24].

**Fig. 6:**
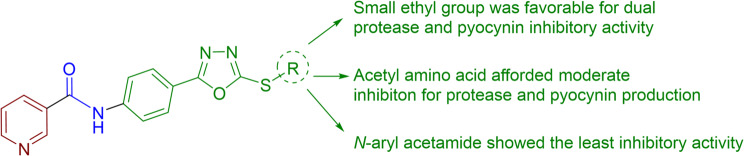
General representation of SAR analysis for the new compounds **3a**,** b**,** 5a**,** b and 7a-i**

It was notable that in both inhibition assays (pyocyanin and protease), the simple alkylated oxadiazole compound 3a and amino acid derivatives 7a-i were preferred over the chlorinated derivatives 5a, b (Fig. 6). From the previous assays, some structure activity relationships (SAR) could be concluded: Among the amino acid derivatives 7a-i, the derivative carrying *β-*alanine (7a) was the most potent pyocyanin inhibitor, while the derivatives carrying *α-alanine*,* glycine and L-tyrosine (*7b, 7c, and 7i respectively) showed the most potent proteolytic activity inhibition. Within the chlorinated derivatives 5a, b, the 4- chloro derivative (5b) was more potent than the 3- chloro derivative (4a) in both inhibition assays (pyocyanin and protease). While the simple alkylated oxadiazoles 3a was the most potent inhibitor amongst all the synthesized compounds in this study.

### Growth inhibition effect of sub-MIC of compound 3a

**Fig. 7:**
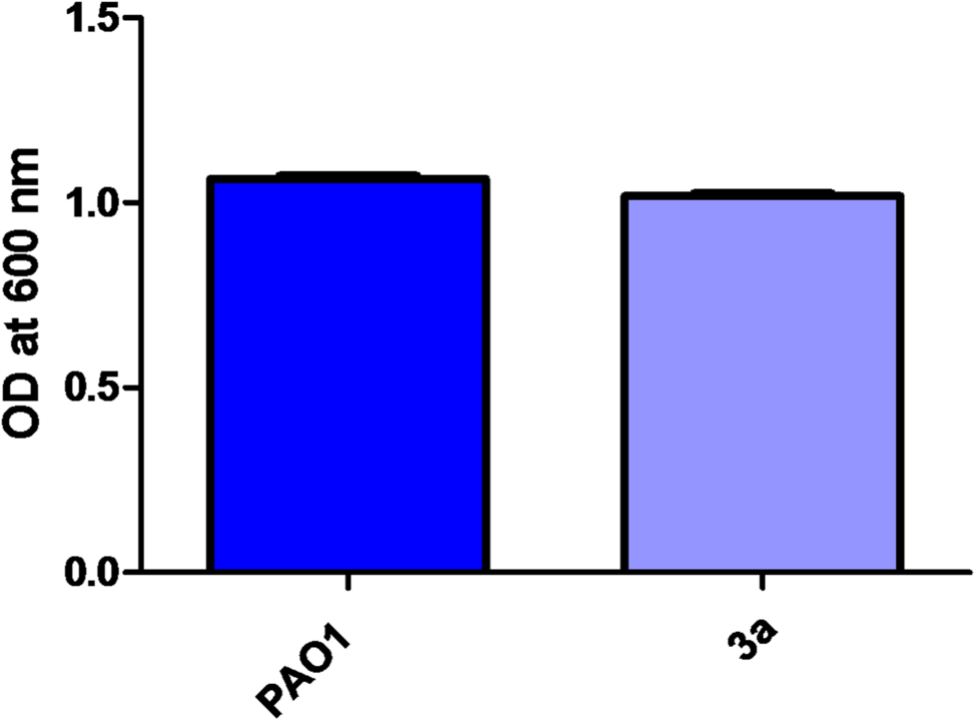
Effect of compound **3a** on PAO1 growth. Compound **3a** showed no statistically significant effect on bacterial growth. Paired t test was used for statistical analysis, *p <* 0.05 was considered significant

Compound 3a was selected for further investigation as being the most active one in screening experiments. The effect of 1/8 MIC of compound 3a on growth of *P. aeruginosa* was tested to ensure that it has no effect on growth. The turbidities of overnight bacterial cultures treated and untreated with compound 3a were measured and there was no statistically significant difference (Fig. 7).

### inhibition of biofilm formation

**Fig. 8:**
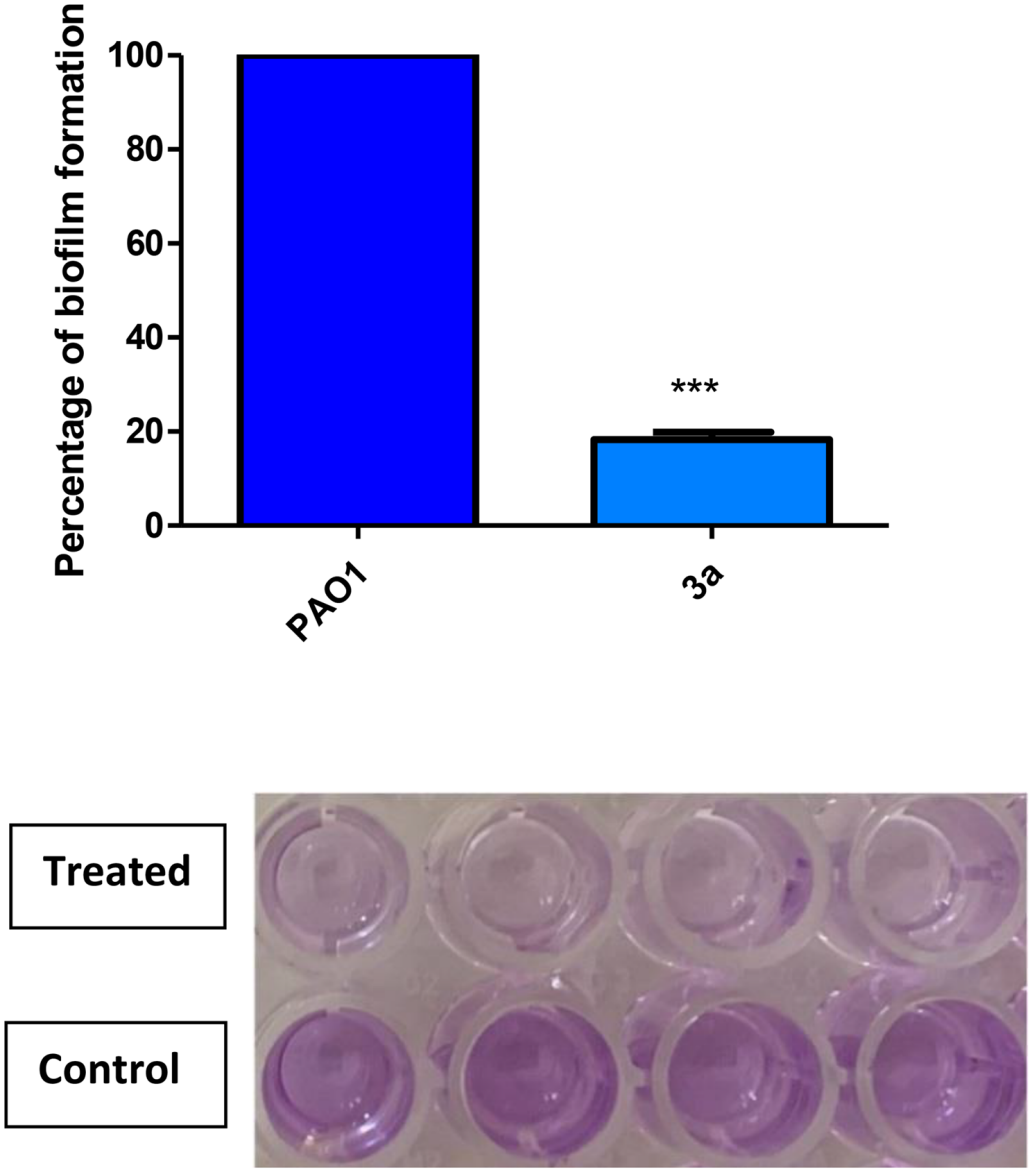
Inhibitory effect of compound **3a** on biofilm formation in *P. aeruginosa*. Compound **3a** significantly reduced biofilm formation. All data are representative of three independent experiments performed in triplicate and expressed as the mean ± SD values in each bar. * *p* < 0.05. Paired t test was used for statistical analysis, *p <* 0.05 was considered significant

Biofilm formation represents an alternative multicellular-like lifestyle to enhance the survival of microbial cells in hostile environment. Furthermore, biofilms enable bacteria to resist the host immunity [31]. The biofilm inhibition assay showed the potent activity of the assessed compound 3a as it reduced biofilm formation by 81.72% (Fig. 8). Compared with the previously reported 1,3,4-oxadiazole derivative which exhibited around 60% inhibition in biofilm formation in PAO1 [24], compound 3a showed superior potency as biofilm inhibitor.

### inhibition of hemolytic activity

**Fig. 9:**
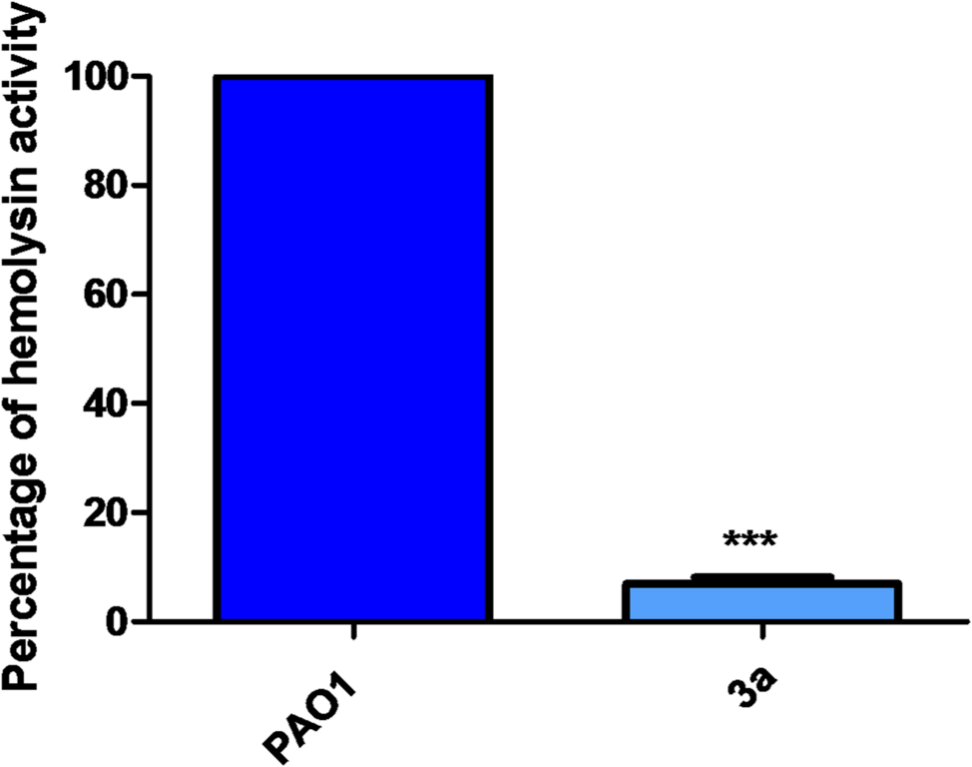
Inhibitory effect of compound **3a** against hemolysin in *P. aeruginosa*. Compound **3a** significantly reduced hemolytic activity. All data are representative of three independent experiments performed in triplicate and expressed as the mean ± SD values in each bar. * *p* < 0.05. Paired t test was used for statistical analysis

Hemolysin is another QS-regulated hydrolytic enzyme with blood cells lysing activity and also it has the ability to resist the host defenses [32]. On quantitative assessment of the hemolytic activity of PAO1 and drug-treated culture supernatants, compound 3a could inhibit hemolytic activity by 93% compared to the control (Fig. 9).

### inhibition of rhamnolipids by compound 3a

**Fig. 10:**
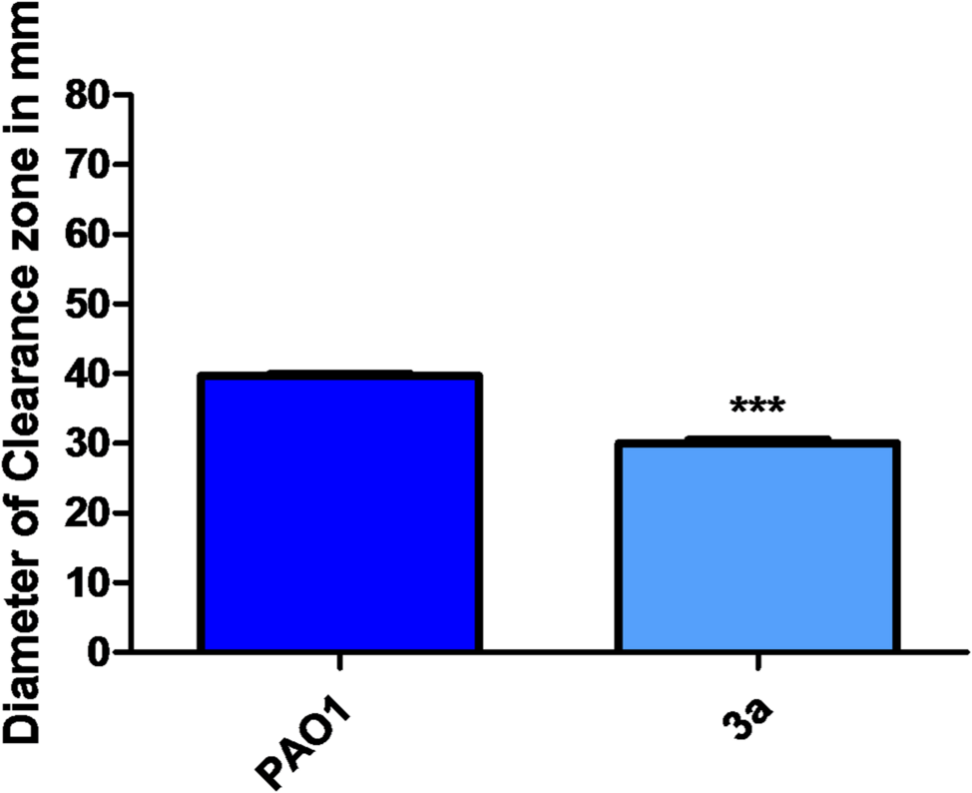
Inhibitory effect of compound **3a** against rhamnolipid production in *P. aeruginosa*. Compound **3a** significantly reduced rhamnolipid production. (Diameter in mm). (All data are representative of three independent experiments performed in triplicate and expressed as the mean ± SD values in each bar. * *p* < 0.05). Unpaired t test was used for statistical analysis

Rhamnolipids are a class of glycolipid produced by *P. aeruginosa* representing a potent virulence factor. Only rhamnolipids are associated with the deterioration of ventilator-associated pneumonia patients [33]. Herein, compound 3a could diminish the rhamnolipid production as shown by the smaller clearance zone produced after the addition of the supernatants of treated cultures to oil compared to that with control PAO1. Compound **3a** decreased the clearance zone by 24.38% (Fig. 10).

### inhibition of swarming motility

**Fig. 11:**
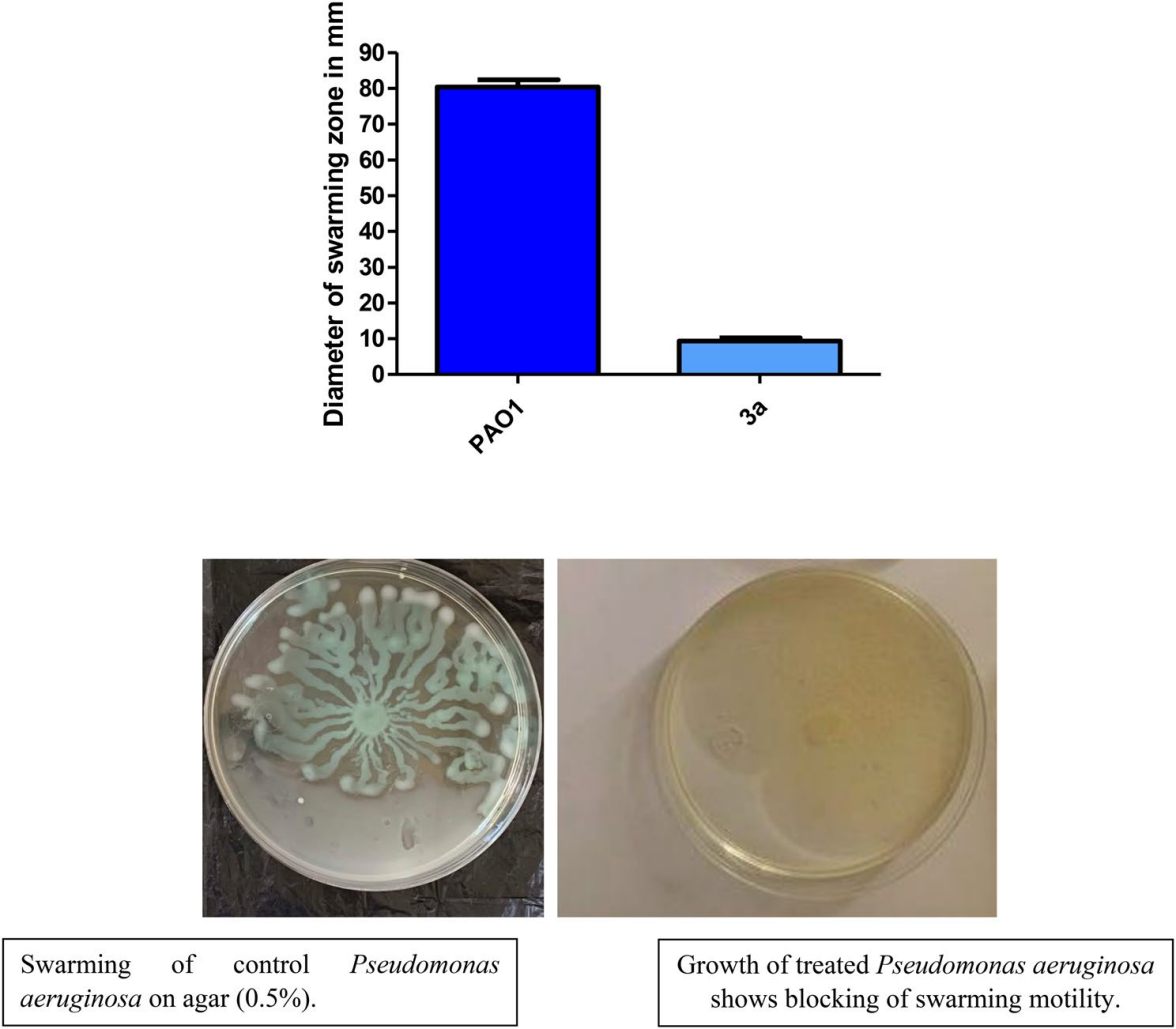
inhibition of swarming motility. The swarming area was reduced by compound **3a.** (Diameter in mm). (All data are representative of three independent experiments performed in triplicate and expressed as the mean ± SD values in each bar. * *p* < 0.05). Unpaired t test was used for statistical analysis

Swarming motility is flagella-dependent rapid multicellular bacterial surface with a vital role in the rapid spread, colonization, and subsequent establishment of bacterial communities [34]. Compound **3a** markedly reduced swarming zone by 88.39% (Fig. 11).

### Molecular modelling

In order to explore the potential bacterial QS inhibition mechanism of our compounds, the most active derivative 3a was docked into the QS receptors (LasR and RhlR).

Docking simulations into *P. aeruginosa* LasR ligand-binding domain bound to its autoinducer (PDB ID: 2UV0) [35] and the modelled RhlR protein (ModelID: b55cde99d7945dee8a458f14d33c17d2) was obtained from ModBase [36]. were initiated by re-docking the co-crystallized ligands; 3-oxo-C12-HSL [*N*-3-oxo-dodecanoyl-l-homoserine lactone] and C4-HSL [*N*-butanoyl-L-homoserine lactone] into their active sites, respectively. The results revealed that the re-docked 3-oxo-C12-HSL and C4-HSL showed 0.6850 Å and 0.5225 Å for RMSD values, and − 11.7697 and − 5.3031 Kcal/mol for scoring, respectively, validating the docking procedure.

**Fig. 12:**
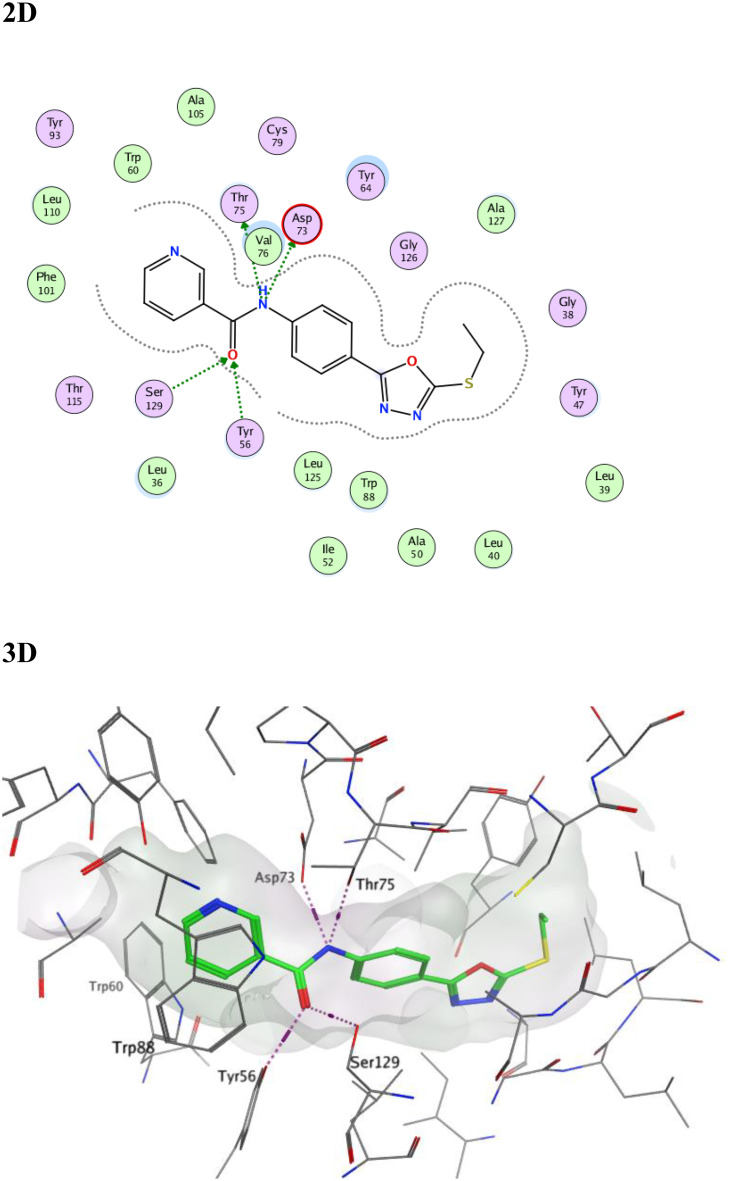
2D and 3D representations of the proposed binding interaction pattern of compound **3a** (green) in the binding site of LasR (PDB ID: 2UV0), S= -9.6849, RMSD = 0.9859

The amino acid residues involved in the LasR autoinducer interactions were Tyr56, Ser129, Trp60, Asp73, Thr75, and Arg71 [35]. The docking score for our compound 3a within LasR active site was − 9.6849 Kcal/mol and it succeeded in the formation of many interactions with the key amino acid residues where its amide carbonyl formed two hydrogen bonds with Tyr56 and Ser129, while its amide nitrogen was involved in additional two hydrogen bonds with Asp73 and Thr75 (Fig. 12). Moreover, the pyridine moiety occupied the same binding region as the lactone head of the native autoinducer with close contacts to the surrounding amino acids making the overall orientation of 3a in LasR active site matched with that of the native autoinducer (Fig. 12).

**Fig. 13:**
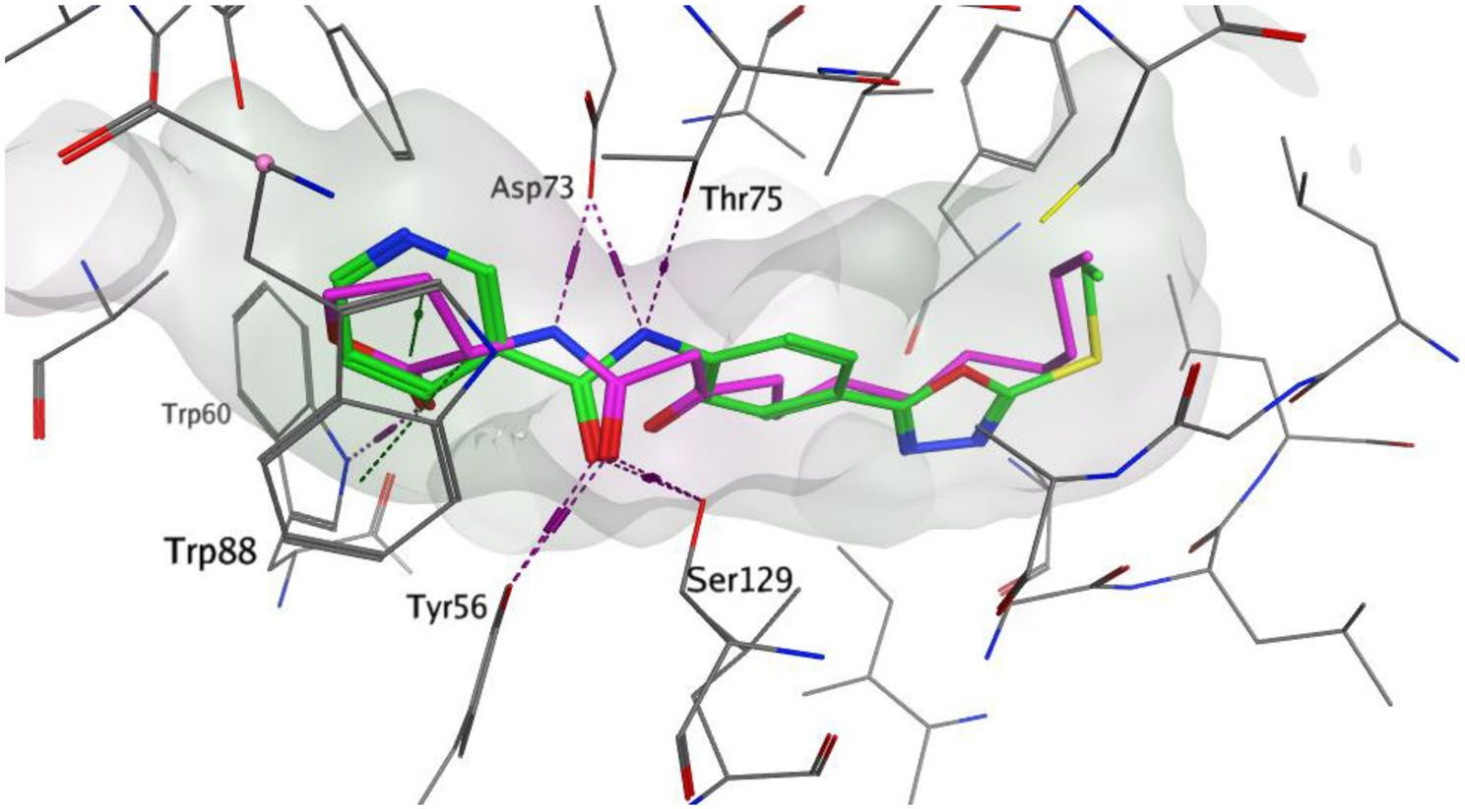
3D representations of compound **3a** (green) superimposed with the co-crystallized auto-inducer (3-oxo-C12-HSL, purple) in the binding site of LasR (PDB ID: 2UV0)

**Fig. 14:**
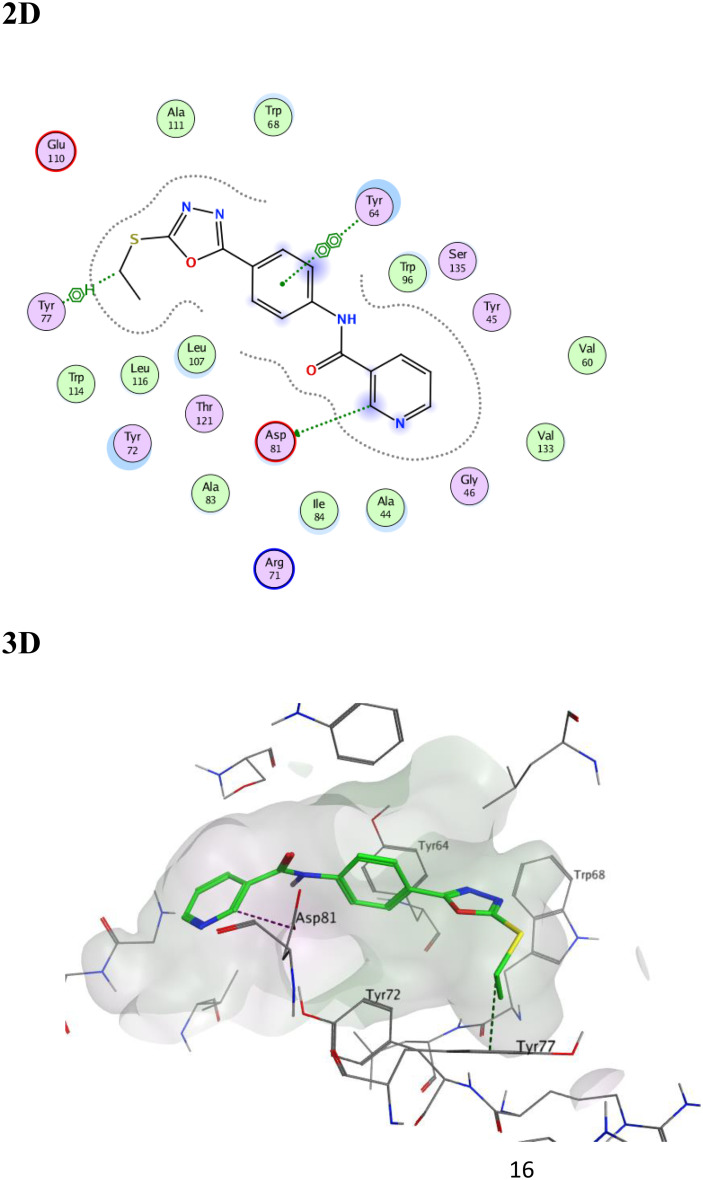
2D and 3D representations of the proposed binding interaction pattern of compound **3a** (green) in the binding site of RhlR, S= -7.4952, RMSD = 0.9580

On the other hand, the amino acid residues involved in the RhlR autoinducer interactions were Trp96, and Asp81. The docking score for our compound **3a** within RhlR active site was − 7.4952 Kcal/mol and it succeeded in the formation of many hydrogen/hydrophobic interactions with the surrounding amino acid residues, where its phenyl ring formed arene-arene interaction with Tyr64 (face-to-face), and its pyridine ring made a hydrogen bond with Asp81. Moreover, its thioethyl made an arene-H interaction with Tyr77. (Fig. 14).

In consistence with our results, Ghameshlouei et al. [28]. have investigated the in-silico effects of 15 synthesized 1, 3, 4-oxadiazole derivatives against *P. aeruginosa*. They demonstrated the inhibitory effect of two generated compounds on the LasR regulatory protein under the control of the QS system in *P. aeruginosa* as demonstrated by molecular docking.

Our docking results revealed that the aforementioned compound 3a had good affinities towards QS receptors (LasR and RhlR) as it showed favored scores and binding interactions with the key amino acid residues within both the LasR and RhlR active sites with higher affinity towards LasR than RhIR active sites (S= -9.6849 vs. -7.4952, respectively). Docking results correlated to the biological results. As 3a showed better docking scores (The lower the score, the stronger the binding affinity of the compound to the active site) and more binding interactions (The more the binding interactions the stronger the binding affinity of the compound to the active site) when docked into LasR active site than when docked into RhIR active site (S= -9.6849 vs. -7.4952, respectively) (Interactions = 4 hydrogen bonds vs. 1 hydrogen bond and 2 hydrophobic bonds, respectively) indicating better affinity and consequently more inhibitory activity against LasR than RhIR.

The observed binding affinity arises from that 3a succeed to occupy the active site as it has the correct size, the correct shape, the favored score and makes favored binding interaction with the key amino acid residue within the active site. As the active site needs to be free and available to exert its action, occupying the active site by 3a will prevent the natural substrate from interacting with its active site and thus 3a exerting its inhibitory activity. The structure of 3a (1,3,4-oxadiazole-nicotinamide hybrid) succeeded into mimicking the natural autoinducer (3-oxo-C12-HSL) orientation within the active site, where the nicotinamide occupied the same binding region as the lactone head of the native autoinducer while phenyl-1,3,4-oxadiazole-S occupied the same binding region as the tail of the native autoinducer (Fig. 13).

## Conclusion

The main objective of this work is to design and synthesize quorum sensing inhibitors that mimic the head and tail structure features of the native auto-inducer of *P. aeruginosa* (AHL). All compounds were tested for their inhibitory activity against protease and pyocyanin production at sub-growth inhibitory concentrations (1/8 MIC). From the tested compounds, the oxadiazole derivative **3a** was the most promising target that reduced both protease activity and pyocyanin production by 38.46% and 70.27% respectively. Moreover, it showed significant reduction of the biofilm formation and hemolysin production by 81.72% and 93% respectively. The Molecular modelling studies explored the binding affinity of compound **3a** toward QS receptors (LasR and RhlR). Therefore, we can conclude that these compounds may serve as potential candidate drug molecules for QS inhibition in *P. aeruginosa*.

## Experimental

### General procedures

All chemical reagents were purchased from commercial sources with the highest purity available. The melting points (^◦^C) of the synthesized compounds were determined in open capillaries using Stuart Melting Point apparatus and are uncorrected. NMR spectra, IR spectra and elemental analyses (C, H, N) were carried out at Applied Nucleic Acid Research Center, Faculty of Sciences, Zagazig University, Zagazig, Egypt. Mass spectra were carried out at the Regional Center of Mycology and Biotechnology, Al-Azhar University, Nasr City, Egypt. The IR-spectra (KBr, cm^− 1^) of the compounds were recorded on Bruker Alpha FT-IR spectrometer. ^1^H NMR and ^13^C APT NMR spectra were recorded on Bruker high performance Digital FT-NMR spectrometer advance III 400 MHz using dimethyl sulfoxide (DMSO)-d_6_ as solvent. Chemical shifts are reported in δ (ppm) relative to the internal tetramethyl silane (TMS) standard. Mass spectra were obtained using a GC/MS Mat 112 S mass spectrometer under EI + ionization technique/mode. Elemental analyses were determined using the Vario MICRO cube (Elementar) CHNS analyzer. All reactions were monitored by thin layer chromatography (TLC) (Rf) on silica gel 60 GF245 (E-Merck, Germany) using an UV lamp for visualization at a wavelength (λ) of 254 nm. Compounds 2, 4a, b and 6a-i were prepared according to reported methods respectively [27, 37–39].

*General procedure for the synthesis of N-(4-(5-(substitutedthio)-1*,*3*,*4-oxadiazol-2- yl)phenyl)nicotinamide (3a*,* b).*

Procedure A: (For compound 3a). A mixture of compound **2** (0.60 g, 2 mmol) and K_2_CO_3_ (0.55 g, 4 mmol) was dissolved in 60 ml water. The solution was filtered followed by addition of diethyl sulfate (0.51 ml, 4mmol) to the filtrate. The solution was stirred at room temperature for 1 h. and the precipitate was filtered off and washed with water and crystalized from ethanol.

*N-(4-(5-(Ethylthio)-1*,*3*,*4-oxadiazol-2-yl) phenyl) nicotinamide (3a)*.

White crystals (69% yield). M.P. 280–282^◦^C. IR (KBr): *ν*_max_ 3419 (NH), 3069 (Ar-H), 2921 (Aliphatic-H), 1681 (C = O), 1602 (C = N), 1500 (C = C) cm^− 1^; ^1^H NMR (400 MHz, DMS0-*d*_6_) δ 10.76 (s, 1H, NH, exchangeable with D_2_0), 9.13 (d, *J =* 1.6 Hz, 1H, pyridine-CH), 8.79 (dd, *J =* 4.8, 1.6 Hz, 1H, pyridine-CH), 8.32 (d, *J =* 8.1 Hz, 1H, pyridine-CH), 8.06–7.95 (m, 4 H, Ar-H), 7.60 (dd, *J =* 8.1, 4.8 Hz, 1H, pyridine-CH), 3.34–3.28 (m, 2 H, CH_2_), 1.43 (t, *J =* 7.3 Hz, 3 H, CH_3_) ppm; ^13^C APT NMR (100 MHz, DMS0-d_6_) δ 164.9 (Ar-C), 164.5 (CO), 163.3 (Ar-C), 152.3 (pyridine-CH), 148.7 (pyridine-CH), 142.1 (Ar-C), 135.6 (pyridine-CH), 130.3 (pyridine-C), 127.2 (Ar-CH), 123.5 (pyridine-CH), 120.4 (Ar-CH), 118.2 (Ar-C), 26.67 (CH_2_), 14.9 (CH_3_) ppm; MS, *m*/*z*: 326.83 (M^+^); Analysis calcd. for C_16_H_14_N_4_O_2_S: C, 58.88; H, 4.32; N, 17.17. Found: C, 58.96; H, 4.46; N, 17.31.

Procedure B: (For compound 3b): To a solution of compound **2** (0.60 g, 2 mmol) and KOH (0.112 g, 2 mmol) in 10 ml ethanol, isopropyl bromide (0.2 ml, 2 mmol) was added. The solution was stirred at room temperature for 24 h. The solution was quenched with ice, the separated solid was filtered off and crystallized from ethanol.

*N-(4-(5-(Isopropylthio)-1*,*3*,*4-0xadiazol-2-yl)phenyl) nicotinamide (3b).*

White crystals (77% yield). M.P. 289–291^◦^C. IR (KBr): *ν*_max_ 3414 (NH), 3066 (Ar-H), 2955 (Aliphatic-H), 1724 C = O), 1603 (C = N), 1473 (C = C) cm^− 1^; ^1^H NMR (400 MHz, DMS0-*d*_6_) δ 10.77 (s, 1H, NH, Exchangeable with D_2_O), 9.13 (d, *J =* 1.6 Hz, 1H, pyridine-CH), 8.79 (dd, *J =* 4.8, 1.6 Hz, 1H, pyridine-CH), 8.32 (d, *J =* 7.9 Hz, 1H, pyridine-CH), 8.04–7.97 (m, 4 H, Ar-H), 7.60 (dd, *J =* 7.9, 4.8 Hz, 1H, pyridine-CH), 3.97–3.87 (m, 1H, CH), 1.47 (d, *J =* 6.8 Hz, 6 H, 2CH_3_) ppm; ^13^C APT NMR (100 MHz, DMS0-d_6_) δ 165.0 (Ar-C), 164.5 (CO), 162.6 (Ar-C), 152.4 (pyridine-CH), 148.8 (pyridine-CH), 142.2 (Ar-C), 135.6 (pyridine-CH), 130.3 (pyridine-C), 127.3 (Ar-CH), 123.6 (pyridine-CH), 120.5 (Ar-CH), 118.2 (Ar-C), 38.4 (CH), 23.1 (2CH_3_) ppm; MS, *m/z*: 340.15 (M^+^); Analysis calcd. for C_17_H_16_N_4_O_2_S: C, 59.98; H, 4.74; N, 16.46. Found: C, 60.11; H, 4.84; N, 16.54.

*General procedure for the synthesis of N-(4-(5-((2-((substituted phenyl) amino)-2-oxoethyl) thio)-1*,*3*,*4-0xadiaz0l-2-yl) phenyl) nicotinamide (5a*,* b).*

To a solution of compound **2** (0.60 g, 2 mmol) and K_2_Co_3_ (0.55 g, 4 mmol) in 20 ml dry acetone, the appropriate *N*-chloroacetylated aromatic amine 4a, b (2 mmol) were added and heated under reflux for 4 h. TLC was used to track the reaction’s progress, and the reaction mixture was concentrated at lower pressure after it was finished. After being washed with water, the precipitate was filtered out and purified by crystallizing in methanol.

*N-(4-(5-((2-((3-Chlorophenyl)amino)-2-oxoethyl)thio)-1*,*3*,*4-oxadiazol-2-yl)phenyl) nicotinamide (5a).*

Yellow crystals (yield 88%). M.P. 247–249^◦^C. IR (KBr): *ν*_max_ 3340 (2NH), 3033 (Ar-H), 2972 (Aliphatic-H), 1667 (C = O), 1644 (C = N), 1518 (C = C) cm^− 1^; ^1^H NMR (400 MHz, DMS0-*d*_6_) δ 10.76 (s, 1H, NH, Exchangeable with D_2_O), 10.63 (s, 1H, NH, Exchangeable with D_2_0), 9.12 (d, *J =* 1.6 Hz, 1H, pyridine-H), 8.79 (dd, *J =* 4.8, 1.6 Hz, 1H, pyridine-H), 8.31 (d, *J =* 7.9 Hz, 1H, pyridine-H), 8.03–7.93 (m, 4 H, Ar-H), 7.80 (s, 1H, Ar-H),7.59 (dd, *J =* 7.9, 4.8 Hz, 1H, pyridine-H), 7.48–7.30 (m, 2 H, Ar-H), 7.15 (d, *J =* 7.9 Hz, 1H, Ar-H), 4.34 (s, 2 H, CH_2_) ppm; ^13^C APT NMR (100 MHz, DMS0-d_6_) δ 165.4 (CO), 165.0 (Ar-C), 164.5 (CO), 162.8 (Ar-C), 152.4 (pyridine-CH), 148.7 (pyridine-CH), 142.2 (Ar-C), 140.0 (Ar-C), 135.6 (pyridine-CH), 133.2 (Ar-C), 130.6 (Ar-CH), 130.3 (pyridine-C), 127.2 (Ar-CH), 123.5 (Ar-CH), 123.4 (pyridine-CH), 120.4 (Ar-CH),118.6 (Ar-CH), 118.0 (Ar-C),117.6 (Ar-CH), 36.8 (CH_2_) ppm; MS, *m/z*: 465.91 (M^+^), 467.46 (M^+^+2); Analysis calcd. for C_22_H_16_ClN_5_O_3_S: C, 56.72; H, 3.46; N, 15.03. Found: C, 56.83; H, 3.62; N, 15.14.

*N-(4-(5-((2-((4-Chlorophenyl)amino)-2-oxoethyl)thio)-1*,*3*,*4-oxadiazol-2-yl)phenyl) nicotinamide (5b).*

Yellow crystals (yield 90%). M.P. 228–230^◦^C. IR (KBr): *ν*_max_ 3344 (2NH), 3030 (Ar-H), 2982 (Aliphatic-H), 1665 (C = O), 1641 (C = N), 1517 (C = C) cm^− 1^; ^1^H NMR (400 MHz, DMS0-*d*_6_) δ 10.76 (s, 1H, NH, Exchangeable with D_2_O), 10.58 (s, 1H, NH, Exchangeable with D_2_0), 9.12 (s, 1H, pyridine-H), 8.78 (d, *J =* 3.8 Hz, 1H, pyridine-H), 8.31 (d, *J =* 7.6 Hz, 1H, pyridine-H), 8.02–7.94 (m, 4 H, Ar-H), 7.66–7.55 (m, 3 H, 2Ar-H and pyridine-H), 7.39 (d, *J =* 8.5 Hz, 2 H, Ar-H), 4.34 (s, 2 H, CH_2_) ppm; ^13^C APT NMR (100 MHz, DMS0-d_6_) δ 165.2 (CO), 165.0 (Ar-C), 164.5 (CO), 162.9 (Ar-C), 152.4 (pyridine-CH), 148.8 (pyridine-CH), 142.2 (Ar-C), 137.6 (Ar-C), 135.6 (pyridine-CH), 130.3 (pyridine-C),128.8(Ar-CH), 127.3(Ar-C), 127.2 (Ar-CH), 123.6 (pyridine-CH), 120.8 (Ar-CH), 120.4 (Ar-CH), 118.1 (Ar-C), 36.8 (CH_2_) ppm; MS, *m/z*: 465.32 (M^+^), 467.81 (M^+^+2); Analysis calcd. for C_22_H_16_ClN_5_O_3_S: C, 56.72; H, 3.46; N, 15.03. Found: C, 56.87; H, 3.58; N, 15.20.

*General procedure for the synthesis of (2-((5-(4-(nicotinamido) phenyl)-1*,*3*,*4-oxadiazol-2-yl) thio) acetyl)-amino acid (7a-i).*

To a solution of compound **2** (0.60 g, 2 mmol) and K_2_CO_3_ (0.55 g, 4 mmol) in 20 ml dry acetone, the appropriate *N*-chloroacetylated amino acids **6a-i** (2 mmol) were added and heated under reflux for 6 h. TLC was used to monitor the reaction’s progress till it was completed then the reaction mixture was concentrated under reduced pressure. The resulting precipitate was dissolved in water and acidified with glacial acetic acid. The precipitate was filtered off, washed with water, and crystallized from methanol.

*(2-((5-(4-(Nicotinamido) phenyl)-1*,*3*,*4-oxadiazol-2-yl) thio) acetyl)-β-alanine (7a)*.

Yellow crystals (yield 85%). M.P. 216–218 ^◦^C. IR: *ν*_max_ 3545 (OH), 3314 (2NH), 3055 (Ar-H), 2960 (Aliphatic-H), 1666 (C = O), 1635 (C = N), 1573 (C = C) cm^− 1^; ^1^H NMR (400 MHz, DMS0-*d*_6_) δ 12.28 (s, 1H, OH, Exchangeable with D_2_O), 10.76 (s, 1H, NH, Exchangeable with D_2_O), 9.13 (s, 1H, pyridine-H), 8.79 (d, *J =* 4.8 Hz, 1H, pyridine-H), 8.42 (s, 1H, NH, Exchangeable with D_2_O), 8.32 (d, *J =* 7.9 Hz, 1H, pyridine-H), 8.09–7.92 (m, 4 H, Ar-H), 7.59 (dd, *J* = 7.9, 4.8 Hz, 1H, pyridine-H), 4.09 (s, 2 H, CH_2_), 3.29 (t, *J* = 6.8 Hz, 2 H, CH_2_), 2.40 (t, *J* = 6.8 Hz, 2 H, CH_2_) ppm; ^13^C APT NMR (100 MHz, DMSO-d_6_) δ 172.7 (CO), 166.1 (CO), 164.9 (Ar-C), 164.5 (CO), 162.9 (Ar-C), 152.3 (pyridine-CH), 148.7 (pyridine-CH), 142.1 (Ar-C), 135.6 (pyridine-CH), 130.3 (pyridine-C), 127.2 (Ar-CH), 123.5 (pyridine-CH), 120.4 (Ar-CH), 118.1 (Ar-C), 35.7 (CH_2_), 35.3 (CH_2_), 33.6 (CH_2_) ppm; MS, *m/z*: 427.65 (M^+^);

Analysis calcd. for C_19_H_17_N_5_O_5_S: C, 53.39; H, 4.01; N, 16.38. Found: C, 53.61; H, 4.12; N, 16.29.

*(2-((5-(4-(Nicotinamido) phenyl)-1*,*3*,*4-oxadiazol-2-yl) thio) acetyl)-α-alanine (7b).*

Pale yellow crystals (yield 78%). M.P. 198–200 ^◦^C. IR (KBr): *ν*_max_ 3550 (OH), 3330 (2NH), 3051 (Ar-H), 2981 Aliphatic-H), 1664 (C = O), 1601 (C = N), 1518 (C = C) cm^− 1^; ^1^H NMR (400 MHz, DMSO-*d*_6_) δ 12.11 (s, 1H, OH, Exchangeable with D_2_O), 10.79 (s, 1H, NH, Exchangeable with D_2_O), 9.13 (s, 1H, pyridine-H), 8.78 (d, *J =* 4.8 Hz, 1H, pyridine-H), 8.62 (d, *J* = 7.0 Hz, 1H, NH, Exchangeable with D_2_O), 8.32 (d, *J =* 7.9 Hz, 1H, pyridine-H), 8.08–7.95 (m, 4 H, Ar-H), 7.59 (dd, *J* = 7.9, 4.8 Hz, 1H, pyridine-H), 4.25–4.17 (m, 1H, CH), 4.15 (s, 2 H, CH_2_), 1.27 (d, *J* = 7.2 Hz, 3 H, CH_3_) ppm; ^13^C APT NMR (100 MHz, DMSO-d_6_) δ 173.7 (CO), 165.7 (CO), 164.9 (Ar-C), 164.5 (CO), 162.8 (Ar-C), 152.3 (pyridine-CH), 148.8 (pyridine-CH), 142.1 (Ar-C), 135.6 (pyridine-CH), 130.3 (pyridine-C), 127.2 (Ar-CH), 123.5 (pyridine-CH), 120.4 (Ar-CH), 118.1 (Ar-C), 48.3 (CH), 35.5 (CH_2_), 17.5 (CH_3_) ppm; MS, *m/z*: 427.23 (M^+^); Analysis calcd. for C_19_H_17_N_5_O_5_S: C, 53.39; H, 4.01; N, 16.38. Found: C, 53.43; H, 4.13; N, 16.42.

*(2-((5-(4-(Nicotinamido) phenyl)-1*,*3*,*4-oxadiazol-2-yl) thio) acetyl) glycine (7c).*

White crystals (yield 80%). M.P. 183–185 ^◦^C. IR (KBr): *ν*_max_ 3540 (OH), 3340 (2NH), 3120 (Ar-H), 2910 Aliphatic-H), 1667 (C = O), 1624 (C = N), 1396 (C = C) cm^− 1^; ^1^H NMR (400 MHz, DMSO-*d*_6_) δ 12.59 (s, 1H, OH, Exchangeable with D_2_O), 10.77 (s, 1H, NH, Exchangeable with D_2_O), 9.13 (d, *J* = 1.7 Hz, 1H, pyridine-H), 8.79 (d, *J =* 4.8, 1.7 Hz, 1H, pyridine-H), 8.63 (t, *J* = 5.7 Hz, 1H, NH, Exchangeable with D_2_O), 8.32 (d, *J =* 7.9 Hz, 1H, pyridine-H), 8.07–7.95 (m, 4 H, Ar-H), 7.59 (dd, *J* = 7.9, 4.8 Hz, 1H, pyridine-H), 4.17 (s, 2 H, CH_2_),3.81 (d, *J* = 5.7 Hz, 2 H, CH_2_) ppm. ^13^C APT NMR (100 MHz, DMSO-d_6_) δ 170.8 (CO), 166.4 (CO), 164.9 (Ar-C), 164.5 (CO), 162.8 (Ar-C), 152.3 (pyridine-CH), 148.7 (pyridine-CH), 142.1 (Ar-C), 135.6 (pyridine-CH), 130.3 (pyridine-C), 127.3 (Ar-CH), 123.5 (pyridine-CH), 120.4 (Ar-CH), 118.1 (Ar-C), 41.2 (CH_2_), 35.4 (CH_2_) ppm; MS, *m/z*: 413.35 (M^+^); Analysis calcd. for C_18_H_15_N_5_O_5_S C, 52.30; H, 3.66; N, 16.94. Found: C, 52.49; H, 3.73; N, 17.06.

*(2-((5-(4-(Nicotinamido) phenyl)-1*,*3*,*4-oxadiazol-2-yl) thio) acetyl)-L-valine (7d).*

White crystals (yield 83%). M.P. 241–243 ^◦^C. IR: *ν*_max_ 3539 (OH), 3274 (2NH), 2937 (Aliphatic-H), 1663 (C = O), 1604 (C = N), 1533 (C = C) cm^− 1^; ^1^H NMR (400 MHz, DMSO-*d*_6_) δ 12.71 (s, 1H, OH, Exchangeable with D_2_O), 10.76 (s, 1H, NH, Exchangeable with D_2_O), 9.13 (d, *J* = 1.6 Hz, 1H, pyridine-H), 8.79 (d, *J =* 4.8, 1.6 Hz, 1H, pyridine-H), 8.49 (d, *J* = 8.4 Hz, 1H, NH, Exchangeable with D_2_O), 8.32 (d, *J =* 8.1 Hz, 1H, pyridine-H), 8.09–7.95 (m, 4 H, Ar-H), 7.59 (dd, *J* = 8.1, 4.8 Hz, 1H, pyridine-H), 4.28–4.15 (m, 3 H, CH and CH_2_), 2.12–2.02 (m, 1H, CH), 0.91 − 0.84 ( m, 6 H, 2CH_3_) ppm; ^13^C APT NMR(100 MHz, DMSO-d_6_) δ 172.5 (CO), 166.4 (CO), 164.9 (CO), 164.5 (Ar-C), 162.8 (Ar-C), 152.3 (pyridine-CH), 148.7 (pyridine-CH),142.1 (Ar-C), 135.6 (pyridine-CH), 130.3 (pyridine-C), 127.2 (Ar-CH), 123.5 (pyridine-CH), 120.4 (Ar-CH), 118.1 (Ar-C), 57.6 (CH), 36.9 (CH_2_), 30.0 (CH), 19.0 (CH_3_), 17.73(CH_3_) ppm; MS, *m/z*: 456.21 (M^+^+1); Analysis calcd. for C_21_H_21_N_5_O_5_S: C, 55.38; H, 4.65; N, 15.38. Found: C, 55.52; H, 4.75; N, 15.46.

*(2-((5-(4-(Nicotinamido) phenyl)-1*,*3*,*4-oxadiazol-2-yl) thio) acetyl)-L-leucine (7e).*

White crystals (yield 79%). M.P. 274–276 ^◦^C. IR (KBr): *ν*_max_ 3548 (OH), 3114 (2NH), 2980, 2957 Aliphatic-H), 1667 (C = O), 1607 (C = N), 1574 (C = C) cm^− 1^; ^1^H NMR (400 MHz, DMSO-*d*_6_) δ 12.25 (s, 1H, OH, Exchangeable with D_2_O), 10.76 (s, 1H, NH, Exchangeable with D_2_O), 9.13 (d, *J* = 1.6 Hz, 1H, pyridine-H), 8.79 (d, *J =* 4.8, 1.6 Hz, 1H, pyridine-H), 8.51 (d, *J* = 8.4 Hz, 1H, NH, Exchangeable with D_2_O), 8.32 (d, *J =* 8.1 Hz, 1H, pyridine-H), 8.12–7.94 (m, 4 H, Ar-H), 7.59 (dd, *J* = 8.1, 4.8 Hz, 1H, pyridine-H), 4.26–4.13 (m, 2 H, CH_2_), 1.83–1.75 (m, 1H, CH), 1.42–1.33 (m, 1H, CH), 1.25–1.12 (m, 2 H, CH_2_), 0.88–0.78 ( m, 6 H, 2CH_3_) ppm. ^13^C APT NMR(100 MHz, DMSO-d_6_) δ 172.6 (CO), 166.3 (CO), 164.9 (CO),164.5 (Ar-C), 162.2 (Ar-C), 152.4 (pyridine-CH), 148.8 (pyridine-CH), 142.1 (Ar-C), 135.6 (pyridine-CH), 130.3 (pyridine-C), 127.3 (Ar-CH), 123.6 (pyridine-CH), 120.4 (Ar-CH), 118.1 (Ar-C), 56.8 (CH), 35.5 (CH_2_), 24.6 (CH_2_), 21.0 (CH), 15.5 (CH_3_), 11.3(CH_3_) ppm; MS, *m/z*: 469.82 (M^+^); Analysis calcd. for C_22_H_23_N_5_O_5_S: C, 56.28; H, 4.94; N, 14.92. Found: C, 56.38; H, 4.98; N, 15.02.

*(2-((5-(4-(Nicotinamido) phenyl)-1*,*3*,*4-oxadiazol-2-yl) thio) acetyl)-L-isoleucine (7f).*

White crystals (yield 82%). M.P. 253–255 ^◦^C. IR (KBr): *ν*_max_ 3470 (OH), 3209 (2NH), 2969, 2936, 1670, 1617, 1514 cm^− 1^; ^1^H NMR (400 MHz, DMSO-*d*_6_) δ 12.65 (s, 1H, OH, Exchangeable with D_2_O), 10.76 (s, 1H, NH, Exchangeable with D_2_O), 9.13 (d, *J* = 1.6 Hz, 1H, pyridine-H), 8.79 (dd, *J =* 4.8, 1.6 Hz, 1H, pyridine-H), 8.61 (d, *J* = 7.9 Hz, 1H, NH, Exchangeable with D_2_O), 8.32 (d, *J =* 8.1 Hz, 1H, pyridine-H), 8.12–7.95 (m, 4 H, Ar-H), 7.60 (dd, *J* = 8.1, 4.8 Hz, 1H, pyridine-H), 4.28–4.08 (m, 3 H, CH and CH_2_), 1.66–1.46 (m, 3 H, CH and CH_2_), 0.84 (d, *J* = 6.5 Hz, 3 H, CH_3_), 0.82–0.79 (m, 3 H, CH_3_) ppm. ^13^C APT NMR (100 MHz, DMSO-d_6_) δ 173.7(CO), 172.0 (CO), 166.2 (CO), 164.6 (Ar-C), 162.8 (Ar-C), 152.4 (pyridine-CH), 148.8 (pyridine-CH),142.1 (Ar-C), 135.6 (pyridine-CH), 130.3 (pyridine-C), 127.3 (Ar-CH), 123.6 (pyridine-CH), 120.4 (Ar-CH),118.1 (Ar-C), 50.8 (CH), 35.5 (CH_2_), 24.2 (CH_2_), 22.8 (CH), 21.2 (CH_3_), 21.1(CH_3_) ppm; MS, *m/z*: 469.96 (M^+^); Analysis calcd. for C_22_H_23_N_5_O_5_S: C, 56.28; H, 4.94; N, 14.92. Found: C, 56.41; H, 4.97; N, 14.99.

*(2-((5-(4-(Nicotinamido) phenyl)-1*,*3*,*4-oxadiazol-2-yl) thio) acetyl)-L-methionine (7 g).*

Pale yellow crystals (yield 71%). M.P. 227–229 ^◦^C. IR (KBr): *ν*_max_ 3308 (OH), 3163 (2NH), 2914 (Aliphatic-H), 1667 (C = O), 1607 (C = N), 1508 (C = C) cm^− 1^; ^1^H NMR (400 MHz, DMSO-*d*_6_) δ 13.45 (s, 1H, OH, Exchangeable with D_2_O), 10.79 (s, 1H, NH, Exchangeable with D_2_O), 9.13 (d, *J* = 1.5 Hz, 1H, pyridine-H), 8.78 (d, *J =* 4.8, 1.5 Hz, 1H, pyridine-H), 8.62 (d, *J* = 7.7 Hz, 1H, NH, Exchangeable with D_2_O), 8.32 (d, *J =* 8.0 Hz, 1H, pyridine-H), 8.06–7.94 (m, 4 H, Ar-H), 7.59 (dd, *J* = 8.0, 4.8 Hz, 1H, pyridine-H), 4.35–4.29 (m, 1H, CH), 4.22–4.12 (m, 2 H, CH_2_), 2.49–2.40 (m, 2 H, CH_2_), 2.01 (s, 3 H, CH_3_), 1.90–1.80 ( m, 2 H, CH_2_) ppm. ^13^C APT NMR (100 MHz, DMSO-d_6_) δ 172.9 (CO), 166.2 (CO), 164.9 (Ar-C), 164.5 (CO), 162.8 (Ar-C), 152.4 (pyridine-CH), 148.8 (pyridine-CH), 142.1 (Ar-C), 135.6 (pyridine-CH), 130.3 (pyridine-C), 127.3 (Ar-CH), 123.5 (pyridine-CH), 120.4 (Ar-CH), 118.1 (Ar-C), 51.7 (CH), 35.5 (CH_2_), 30.9 (CH_2_), 29.6 (CH_2_), 14.52(CH_3_) ppm; MS, *m/z*: 487.55 (M^+^); Analysis calcd. for C_21_H_21_N_5_O_5_S_2_: C, 51.73; H, 4.34; N, 14.36. Found: C, 51.86; H, 4.41; N, 14.52.

*(2-((5-(4-(Nicotinamido) phenyl)-1*,*3*,*4-oxadiazol-2-yl) thio)acetyl)-L-phenylalanine (7 h).*

White crystals (yield 77%). M.P. 162 − 146 ^◦^C. IR (KBr): ): *ν*_max_ 3450 (OH), 3340 (2NH), 3065 (Ar-H), 2963 (Aliphatic-H), 1667 (C = O), 1625 (C = N), 1563(C = C) cm^− 1^; ^1^H NMR (400 MHz, DMSO-*d*_6_) δ 12.18(s, 1H, OH, Exchangeable with D_2_O), 10.77 (s, 1H, NH, Exchangeable with D_2_O), 9.13 (d, *J* = 1.7 Hz, 1H, pyridine-H), 8.78 (dd, *J =* 4.8, 1.7 Hz, 1H, pyridine-H), 8.66 (d, *J* = 7.8 Hz, 1H, NH, Exchangeable with D_2_O), 8.32 (d, *J =* 7.9 Hz, 1H, pyridine-H), 8.04–7.96 (m, 4 H, Ar-H), 7.59 (dd, *J* = 7.9, 4.8 Hz, 1H, pyridine-H),7.28–7.16 (m, 5 H, Ar-H), 4.48–4.42 (m, 1H, CH), 4.15–4.08 (m, 2 H, CH_2_), 3.07 (dd, *J* = 13.8, 5.1 Hz, 1H, C*H*_*A*_H_B_), 2.90 (dd, *J* = 13.8, 8.5 Hz, 1H, CH_A_*H*_*B*_) ppm. ^13^C APT NMR (100 MHz, DMSO-d_6_) δ 172.5 (CO), 166.1 (CO), 164.9 (Ar-C), 164.6 (CO), 162.8 (Ar-C), 152.4 (pyridine-CH), 148.8 (pyridine-CH), 142.1 (Ar-C), 137.3 (Ar-C), 135.6 (pyridine-CH), 130.3 (pyridine-C),129.1(Ar-CH), 128.2 (Ar-CH), 127.3 (Ar-CH), 126.5 (Ar-CH), 123.6 (pyridine-CH), 120.4 (Ar-CH), 118.2 (Ar-C), 54.0 (CH), 36.8 (CH_2_), 35.4 (CH_2_) ppm; MS, *m/z*: 519.96 (M^+^); Analysis calcd. for C_25_H_21_N_5_O_5_S: C, 59.63; H, 4.20; N, 13.91. Found: C, 59.59; H, 4.28; N, 14.08.

*(2-((5-(4-(Nicotinamido) phenyl)-1*,*3*,*4-oxadiazol-2-yl) thio)acetyl)-L-tyrosine (7i).*

White crystals (yield 74%). M.P. 179–181 ^◦^C. IR (KBr): *ν*_max_ 3539 (OH), 3274 (2NH), 3074 Ar-H), 2976 (Aliphatic-H), 1665 (C = O), 1637 (C = N), 1504 (C = C) cm^− 1^; ^1^H NMR (400 MHz, DMSO-*d*_6_) δ 12.79 (s, 1H, OH, Exchangeable with D_2_O), 10.76 (s, 1H, NH, Exchangeable with D_2_O), 9.21 (s, 1H, OH, Exchangeable with D_2_O), 9.13 (d, *J* = 1.6 Hz, 1H, pyridine-H), 8.78 (dd, *J =* 4.8, 1.6 Hz, 1H, pyridine-H), 8.62 (d, *J* = 7.9 Hz, 1H, NH, Exchangeable with D_2_O), 8.32 (d, *J =* 8.0 Hz, 1H, pyridine-H), 8.06–7.94 (m, 4 H, Ar-H), 7.59 (dd, *J* = 8.0, 4.8 Hz, 1H, pyridine-H), 7.00 (d, *J* = 8.4 Hz, 2 H, Ar-H), 6.64 (d, *J* = 8.4 Hz, 2 H, Ar-H), 4.40–4.36 (m, 1H, CH), 4.19–4.08 (m, 2 H, CH_2_), 2.94 (dd, *J* = 13.9, 5.2 Hz, 1H, *CH*_*A*_H_B_), 2.78 (dd, *J* = 13.9, 8.2 Hz, 1H, CH_A_*H*_*B*_) ppm. ^13^C APT NMR (100 MHz, DMSO-d_6_) δ 172.6 (CO), 166.1 (CO), 164.9 (CO),164.5 (Ar-C), 162.8 (Ar-C), 156.0 (Ar-C), 152.4 (pyridine-CH), 148.8 (pyridine-CH),142.1 (Ar-C), 135.6 (pyridine-CH), 130.3 (pyridine-C),130.1(Ar-CH), 127.2 (Ar-CH), 127.17 (Ar-C), 123.6 (pyridine-CH), 120.4 (Ar-CH),118.2 (Ar-C), 115.0 (Ar-CH), 54.3 (CH), 36.0 (CH_2_), 35.5 (CH_2_) ppm; MS, *m/z*: 503.68 (M^+^); Analysis calcd. For C_25_H_21_N_5_O_6_S: C, 57.80; H, 4.07; N, 13.48. Found: C, 57.93; H, 4.12; N, 3.64.

### Microbiology

#### Media and chemicals

Tryptone, Luria-Bertani (LB) broth, LB agar were purchased from Lab M Limited, Lancashire, United Kingdom, Tryptone soya broth, Mueller Hinton broth, Mueller Hinton agar were obtained from Oxoid, Hampshire, UK, while. Dimethyl sulphoxide (DMSO) was obtained from Sigma (St. Louis, USA).

#### *Bacterial strains*

*P. aeruginosa* PAO1 standard strain strain was kindly gifted by the Department of Microbiology, Faculty of Pharmacy, Mansoura University. This strain was originally isolated from wound infection.

#### Determination of minimum inhibitory concentration (MIC)

The broth microdilution method was used in which two-fold serial dilutions of tested compounds (from 40 to 0.312 mg/ml) were made in Mueller-Hinton broth. These dilutions were added in aliquots of 100 µl to the wells of microtiter plate. Then, similar volumes of PAO1 suspension in Mueller-Hinton broth (cell density of 1 × 10^6^ CFU/ml) were added to the wells. The microtiter plate was incubated at 37 °C for 20 h, and the lowest concentration of each compound that inhibited visible growth was determined as the minimum inhibitory concentration [40].

#### Screening for protease inhibition

To investigate if the tested compounds were able to inhibit the proteolytic activity of PAO1, the skim milk agar method was employed. Overnight LB cultures of *P. aeruginosa* PAO1 either with or without 1/8 MIC of each compound were prepared and then centrifuged at 10,000 rpm for 15 min to obtain the supernatants. The supernatants in aliquots of 100 µl were delivered to the wells in 5% skim milk agar plates. The clear zones around the wells were measured after overnight incubation at 37 °C [41].

### Screening for pyocyanin inhibition

The interference with pyocyanin pigment production by PAO1 when treated with compounds was quantified according to Das and Manefield. Overnight LB PAO1 cultures were prepared and diluted to optical density of 0.4 at 600 nm. One ml LB broth tubes with or without 1/8 MIC of compounds were inoculated with 10 µl of the prepared suspension. The tubes were incubated at 37 °C for 48 h, and then centrifuged at 10,000 rpm for 10 min to separate the supernatants, in which the pyocyanin was quantified at 691 nm by Biotek Spectrofluorometer (Biotek, USA) [42]. The test was made in triplicate.

### Effect of sub-inhibitory concentration of **3a** on bacterial growth

To ensure the lack of the effect of compound **3a** on the growth of PAO1, overnight culture of PAO1 was used to inoculate LB broth or LB broth with compound **3a**. After overnight incubation of LB broth tubes at 37 °C, the turbidities of LB cultures were measured at 600 nm by using Biotek Spectrofluorometer (Biotek, USA) [43]. The test was made in triplicate.

### Biofilm inhibition assay

Overnight culture of PAO1 in TSB was diluted to an approximate cell density of 1 × 10^6^ CFU/ml. Then, 0.1 ml aliquots of the suspension were added into the wells of microtiter plate containing or not containing 1/8 MIC of compound **3a**. The plate was incubated at 37 °C for 24 h and then the non-adherent cells were removed. The wells were washed, and the adherent cells were treated with fixing methanol (99%) for 20 min. The wells were stained with crystal violet (1%) and the excess stain was washed water. Glacial acetic acid (33%) was added to dissolve the dye and the absorbance was measured at 590 nm with Biotek spectrofluorometer (Biotek, USA) [44]. The test was made in triplicate.

### Hemolysin inhibition assay

The reduction in hemolytic activity of PAO1 by compound **3a** was determined according to Rossignol *et* al. To the cell free supernatant (500 µl), 700 µl of fresh 2% saline suspension of erythrocytes were added. The released hemoglobin from lysed RBCs incubated at 37 °C for 2 h was separated by centrifugation at 2500 *g* for 5 min at 4 °C and measured at 540 nm. The hemoglobin released was compared with positive control (SDS to erythrocyte suspension) and negative control (erythrocytes in LB broth) at the same conditions. The hemolytic activity was determined according to Rossignol et al. [45]. The test was made in triplicate.

### Rhamnolipids inhibition assay

The oil displacement method was used [46]. Crude oil (20 µl) was added to distilled (15 ml) H_2_O in a Petri dish. Ten µl aliquots of supernatants of control or treated PAO1 were added to the oil drop and the clearing zones were measured as a measure of the biosurfactant activity of rhamnolipids. The test was made in triplicate.

### Swarming motility inhibition assay

The ability of compound 3a to inhibit swarming motility was investigated using control swarming agar plates with 0.5% agar and swarming agar plates with sub-MIC of compound 3a. PAO1 was overnight incubated in tryptone broth and then diluted. Two µl of diluted cultures were spotted on the surface of agar plate and the plates were incubated at 37 °C for 16 h, after which the swarming zones were measured [47]. The test was made in triplicate.

### Statistical analysis

Unpaired and paired t tests, Graph Pad Prism 5 was used to investigate the significance of the inhibitory activities of compounds against swimming motility, growth, biofilm formation, and rhamnolipid. One-way ANOVA test followed by Dunnett posttest was used to determine the significance the effects on protease, and pyocyanin. *P* values < 0.05 were considered statistically significant.

### Molecular docking

The X-ray crystal structures of *P. aeruginosa* LasR ligand-binding domain bound to its autoinducer (PDB ID: 2UV0) [35] and the modelled RhlR protein (ID: b55cde99d7945dee8a458f14d33c17d2) [36] were retrieved from the Protein Data Bank (http://www.rcsb.org) [48] and ModBase [36], respectively.The molecular docking studies were done by using the Molecular Operating Environment; MOE 2019.0102 (Chemical Computing Group, Montreal, CA) [49]. The protein structures were prepared using MOE’s quick preparation tool through the Amber10: EHT forcefield. The compound was prepared by energy minimization, adding hydrogen atoms, and calculating partial charges, and potential energies. The co-crystallized ligands were re-docked into their corresponding active site for validation of the docking results through measuring the root mean square deviations (RMSD). The docking protocol is a triangle matcher, using London dG as the initial scoring approach and GBVI/WSA dG as the final scoring approach. The docking energy scores (Score; Kcal/mol) and visual inspection of 2D and 3D poses of the ligand–receptor interactions were used for the analysis of the docking results.

## Data Availability

The 1HNMR and 13CNMR charts of the current study are available from the corresponding author upon reasonable request.

## Abbreviations

AHLs: N-acylated L-homoserine Lactones
AIs: Autoinducers
QS: Quorum Sensing
QSIs: Quorum-Sensing Inhibitors
PCN: Pyocyanin
3-oxo-C12-HSL: N-3-oxo-dodecanoyl-l-Homoserine Lactone
C4-HSL: N-butanoyl-L-Homoserine Lactone
MIC: Minimum Inhibitory Concentration
PDB: Protein Data Bank
MOE: Molecular Operating Environment Software
